# Supplementary material for: Essential gene complement of Planctopirus limnophila from the bacterial phylum Planctomycetes
Source: Nat Commun. 2023 Nov 9;14:7224. doi: 10.1038/s41467-023-43096-3 (PMC10632474; doi:10.1038/s41467-023-43096-3)
Supplement: Supplementary file 1 — Supplementary Information [file 41467_2023_43096_MOESM1_ESM.pdf]

Essential gene complement of *Planctopirus limnophila* from the bacterial phylum *Planctomycetes*

Elena Rivas-Marin[1#$], David Moyano-Palazuelo[1$], Valentina Henriques[1], Enrique Merino[2], Damien P. Devos[1#*]

[1] Centro Andaluz de Biología del Desarrollo, CSIC, Universidad Pablo de Olavide, Seville, Spain.

[2] Instituto de Biotecnología, Universidad Nacional Autónoma de México, Cuernavaca, Morelos, México.

#Addresses for correspondence to ERM (erivmar@upo.es) and DPD (damienpdevos@gmail.com).

$ Both authors contributed equally.

* Present address: Institut Pasteur de Lille, Centre d'Infection et d'Immunité de Lille, University of Lille, Lille, France.

This PDF file includes:

a) **Supplementary Tables S1 to S4.**
b) **Supplementary Figures S1 to S5.**

a) Supplementary Tables

Supplementary Table 1. LPS and outer membrane genes.

**Gene name**	**COG**	**Locus tag**	**Essentiality**
lpxA	COG1043	Plim_0918	Essential
lpxB	COG0763	Plim_0943	Essential
lpxC	COG0774	Plim_0919	Essential
lpxD	COG1044	Plim_2360	Essential
lpxK	COG1663	Plim_0158	Essential
lpxL	COG1560	Plim_1862	Essential
lpxG	COG1408	Plim_2910	Non-essential
lpxH	COG2908	Plim_0044	Non-essential
bamA	COG4775	Plim_2365	Essential
bamB	COG1520	Plim_3592, Plim_4240, Plim_1699, Plim_3506, Plim_2630, Plim_4033, Plim_4007, Plim_3852, Plim_0945, Plim_2941, Plim_2943, Plim_2944, Plim_0982, Plim_0221, Plim_1978, Plim_1930, Plim_4104, Plim_0234, Plim_1863, Plim_0258, Plim_0250, Plim_1848, Plim_2341, Plim_0777, Plim_3570, Plim_2376, Plim_0598, Plim_2733, Plim_2782	-
bamC	COG3317	-	-
bamD	COG4105	Plim_0726, Plim_3586	Essential

lgt	COG0682	Plim_2253	Essential
lnt	COG0815	Plim_3232	Essential
secB	COG1952	-	-
secG	COG1314	Plim_2050	Non-essential
secY	COG0496	Plim_0496	Essential
secE	COG0690	Plim_0437	Essential

Supplementary Table 2. Strains used in this work.

**Strain name**	**Genotype**	**Reference**
Escherichia coli DH5α	F^- φ80*lacZ*ΔM15 Δ(*lacZYA-argF)U169 recA1 endA1 hsdR17*($r_K^-m_K^-$) *supE44 thi-1 gyrA relA1*	(Hanahan, 1983)[1]
E. coli ER2566	B F^- λ^- *fhuA2* [*lon*] *ompT lacZ*::T7*.1 gal sulA11* Δ(*mcrC-mrr*)*114*::IS*10* R(*mcr-73*::miniTn*10*)(Tet^S)2 R(*zgb-210*::Tn*10*)(Tet^S) *endA1* [*dcm*]	New England Biolab
Planctopirus limnophila DSM3776	Wild type strain	(Hirsch, 1985)[2]
P. limnophila DV051	Δ*mraY1*. Km^R.	This work
P. limnophila DV053	Δ*murJ*. Gm^R.	This work
P. limnophila DV054	Δ*pbp2*. Km^R.	This work
P. limnophila DV057	Δ*mraY2*. Km^R	This work
P. limnophila DV059	Δ*ftsQ*. Km^R.	This work
P. limnophila DV078	Δ*rodA*. Gm^R	This work
P. limnophila DV079	Δ*mraW1*. Km^R.	This work
P. limnophila DV080	Δ*mraW2*. Gm^R.	This work

Supplementary Table 3. Plasmids used in this work.

**Plasmid name**	**Main features**	**Source**
pTXB1-Tn5	IPTG-inducible expression of Tn*5* transposase fused to Mxe Intein and	Addgene plasmid

	Chitin-binding domain.	60240
pDV087	950 bp upstream and 977 bp downstream of *mraY1* gene from *P. limnophila* flanking a kanamycin resistance gene cloned into pEX18Tc. $Km^{R,}$ Tc^{R}.	This work
pDV088	1101 bp upstream and 1044 bp downstream of *pbp2* gene from *P. limnophila* flanking a kanamycin resistance gene cloned into pEX18Tc. $Km^{R,}$ Tc^{R}.	This work
pDV089	1068 bp upstream and 1054 bp downstream of *murJ* gene from *P. limnophila* flanking a gentamicin resistance gene cloned into pEX18Tc. Gm^{R}, Tc^{R}.	This work
pDV092	912 bp upstream and 893 bp downstream of *mraY2* gene from *P. limnophila* flanking a kanamycin resistance gene cloned into pEX18Tc. $Km^{R,}$ Tc^{R}.	This work
pDV096	790 bp upstream and 801 bp downstream of *ftsQ* gene from *P. limnophila* flanking a kanamycin resistance gene cloned into pEX18Tc. $Km^{R,}$ Tc^{R}.	This work
pDV135	808 bp upstream and 819 bp downstream of *rodA* gene from *P. limnophila* flanking a gentamicin resistance gene cloned into pEX18Tc. Gm^{R}, Tc^{R}.	This work
pDV140	736 bp upstream and 742 bp downstream of *mraW1* gene from *P. limnophila* flanking a kanamycin resistance gene cloned into pEX18Tc. $Km^{R,}$ Tc^{R}.	This work
pDV141	736 bp upstream and 742 bp downstream of *mraW2* gene from *P. limnophila* flanking a gentamicin resistance gene cloned into pEX18Tc. Gm^{R}, Tc^{R}.	This work

Supplementary Table 4: Oligonucleotides used in this work. Underlined restriction sites.

**Primer name**	**Primer sequence**	**Target**
Left MraY1 limno fw	CATGAGCTCGTATGTCGAAGACAACGCTCC	MraY1 upstream region
Left MraY1 limno rv	TGAGGATCCCCTTGCTGCGATCCCTCTG	MraY1 upstream region
Right MraY1limno fw	TGAGGATCCATACTCATGGTGAGAAATTGCC	MraY1 downstream region
Right MraY1limno rv	CATGTCGACGTTCCCACCAGCCCCAGTTC	MraY1 downstream region
Left PBP2 limno fwd	TCATGAATTCGCCGAAAGTAGATTTCGCTCC	PBP2 upstream region
Left PBP2 limno rv	TGAGGATCCCACAAGTTTTGGCTAATAGTGG AATC	PBP2 upstream region
Right PBP2 limno fw	TGAGGATCCCTCTTGTTTCAGGTGCCAAG	PBP2 downstream region
Right PBP2 limno rv	CATGTCGACGGAATCCGCATAAGAGGTGC	PBP2 downstream region
Left MraY2 limno fw	CATGAGCTCGGCATCGTCGATTCGATTGAG	MraY2 upstream region
Left MraY2 limno rv	TGAGGATCCTGAGTGATCGACACGATAACAG	MraY2 upstream region
Right MraY2 limno fw	TGAGGATCCATGCCTTCTTTGCGAACGATG	MraY2 downstream region

Right MraY2 limno rv	CATGTCGACGCAAATGTATTCGCCAAAGCG	MraY2 downstream region
Left FtsQ limno fw	TACAGAATTCGCAGATCTTCGTGAGCACCG	FtsQ upstream region
Left FtsQ limno rv	CGAGGATCCCAGAGCCACTCCATCATCAG	FtsQ upstream region
Right FtsQ limno fw	CGAGGATCCAGTTTGCGAGTTTGATGGGTTC	FtsQ downstream region
Right FtsQ limno rv	ACAAAGCTTCAGCGAAAACATCGACTCGAC	FtsQ downstream region
Left RodA limno fw	TGAGAGCTCAGGATCAGATGCCCAAACTG	RodA upstream region
Left RodA limno rv	TTGGGATCCGACGACCTTTGTTACGCAGC	RodA upstream region
Right RodA limno fw	TTGGGATCCTTCGAGGTGCTTGTCAGACG	RodA downstream region
Right RodA limno rv	GTAGTCGACTACCCGTCTTTTTCGAGGTGC	RodA downstream region
Left MraW1 limno fw	TGAGAGCTCTCACCGCTATGAGCGAGTGG	MraW1 upstream region
Left MraW1 limno rv	TTGGGATCCAGCCCGCTGGGACGAGTTTC	MraW1 upstream region
Right MraW1 limno fw	TTGGGATCCGCATTAGAGATCCGGTGCAAAG	MraW1 downstream region
Right MraW1 limno rv	GTAGTCGACATCTGTGGTCTCCTCGCCAC	MraW1 downstream region
Left MraW2 limno fw	TGAGAGCTCATTCTTTGCGGAAAGGTCTTC	MraW2 upstream region
Left MraW2 limno rv	TTGGGATCCGGTCTTCCCATCATACCAAC	MraW2 upstream region
Right MraW2 limno fw	TTGGGATCCACTGATGCGGGGCAATCACG	MraW2 downstream region
Right MraW2 limno rv	GTAGTCGACGACAGGAACATCCGAACTTC	MraW2 downstream region
Km BamHI fwd	GTTGGATCCGCGTCGGCTTGAACGAATTG	Kanamycin resistant gene
Km BamHI rv	TGAGGATCCCATTTCGAACCCCAGAGTCC	Kanamycin resistant gene
Gm BamHI fw	TCAGGATCCGTTGACATAAGCCTGTTCGG	Gentamicin resistant gene
Gm BamHI rv	CATGGATCCTTAGGTGGCGGTACTTGGG	Gentamicin resistant gene
Km IS fwd	*CTGTCTCTTATACACATCT*GCGTCGGCTTGAACGAATTG	Kanamycin resistant gene bearing Tn*5* IS sequence
Km IS rv	*CTGTCTCTTATACACAT*CTCATTTCGAACCCCAGAGTCC	Kanamycin resistant gene bearing Tn*5* IS sequence
Map Tn*5* A fw	ATCAGGACATAGCGTTGGC	Tn*5* transposon
Map Tn*5* b fw	AAGAGCTTGGCGGCGAATG	Tn*5* transposon
CEKG 2A	GGCCACGCGTCGACTAGTACN_{10}AGAG	Random oligonucleotide
CEKG 2B	GGCCACGCGTCGACTAGTACN_{10}ACGCC	Random oligonucleotide
CEKG 2C	GGCCACGCGTCGACTAGTACN_{10}GATAT	Random oligonucleotide
CEKG 4	GGCCACGCGTCGACTAGTAC	CEKG 2B/2C72D

Out DV051 fwd	ATGAGGGCCATTATGGTTCTC	To check DV051 mutant
Out DV051 rv	CTTGTCGAATGAGTTGCAGTC	To check DV051 mutant
Out DV053 fwd	AAGAGTTCCGCAAGTCGTTCG	To check DV053 mutant
Out DV053 rv	ATTCCCGACTTCCTCAGCTG	To check DV053 mutant
Out DV054 fwd	CTTCCTGACAACCTCCAACGC	To check DV054 mutant
Out DV054 rv	CTTCGCGAACCCCCAGCTCG	To check DV054 mutant
Out DV057 fwd	TCTGCAACAGCGAAAAGGGG	To check DV057 mutant
Out DV057 rv	GCATCCTGCACCATTCGCTG	To check DV057 mutant
Out DV059 fwd	AAAGACGTGGCTGATGGTCG	To check DV059 mutant
Out DV059 rv	TGATGGGTGATGGTTCCGTC	To check DV059 mutant
Out DV078 fwd	TATCCCAGGTTCTCCATCCC	To check DV078 mutant
Out DV078 rv	TTCCCAAGCTGATATCGAAC	To check DV078 mutant
Out DV079 fwd	CACAATGGCGACATCTTCCG	To check DV079 mutant
Out DV079 rv	CTATGGCACAGATCATGTTC	To check DV079 mutant
Out DV080 fwd	CGAGCACAGTTGGTACAGC	To check DV080 mutant
Out DV080 rv	GATGAGTTGATACCCCAAGC	To check DV080 mutant

REFERENCES

1. Hanahan, D. Studies on transformation of *Escherichia coli* with plasmids. *J. Mol. Biol.* 166, 557–580 (1983).
2. Hirsch, P. & Müller, M. *Planctomyces limnophilus* sp. nov., a Stalked and Budding Bacterium from Freshwater. *Systematic and Applied Microbiology* 6, 276–280 (1985).

b) Supplementary Figures

Figure S1. Insertion index distribution in *P. limnophila.* The red curve represents the fit of the left part of this distribution to an exponential distribution. The blue curve represents the fit of the right-hand side to a gamma distribution. The green and orange lines represent the cut-offs used for the fits of the distributions. The histogram and curves represent probability densities, with the histogram normalized such that the total area under the bars is 1.

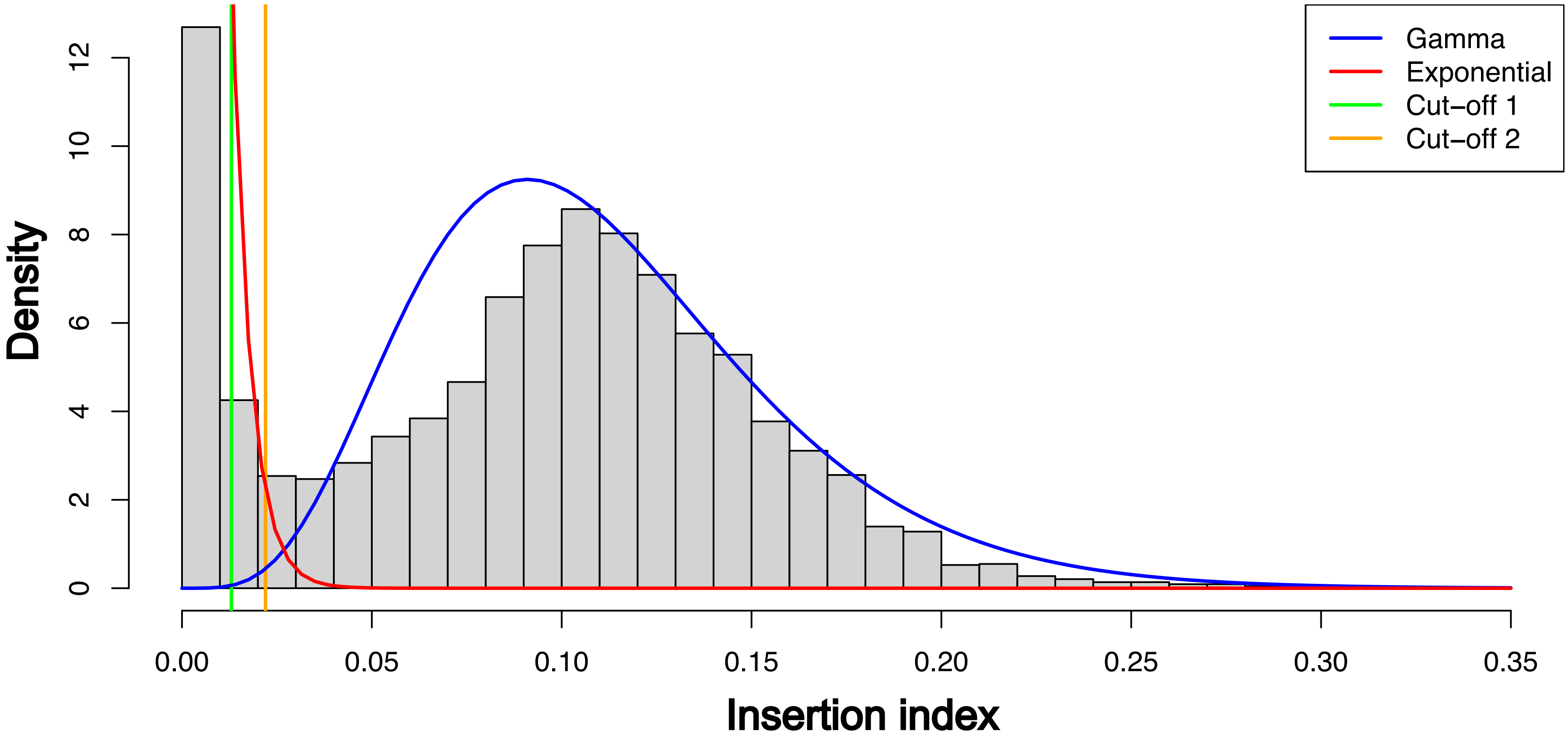

Figure S2. Visualization of "domain essential" proteins. The vertical lines represents the transposon insertion sites in each of the represented proteins. Pfam protein domains are represented in different colors with their corresponding Pfam ID. The X-axis represents the protein length in amino acids. a) Proteins reported as unclear. b) Proteins reported as non-essential.

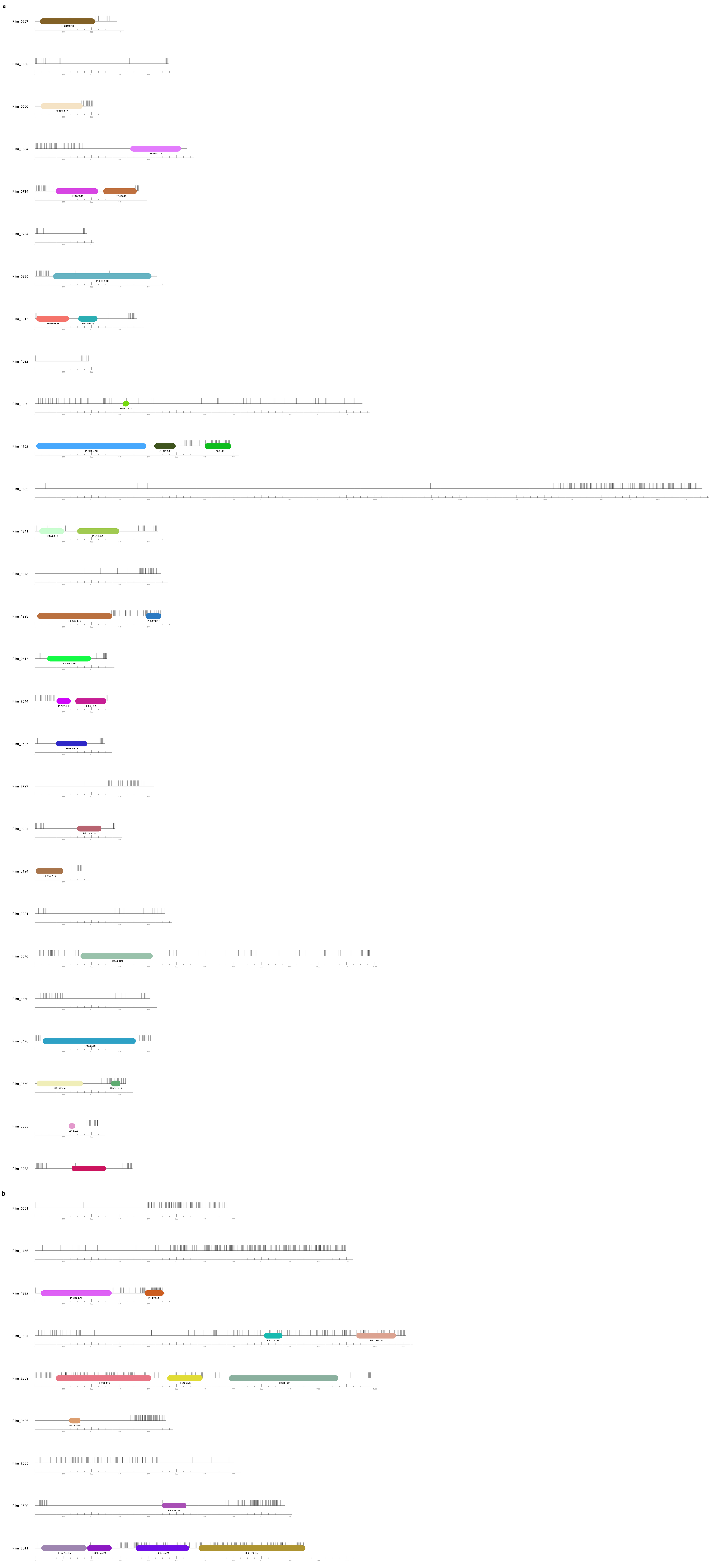

Figure S3. Complete phyletic distribution of *P. limnophila* essential genes. NOG heatmap according to the EggNOG database annotations for the *P. limnophila* essential gene set. Each row represents the essential genes reported in this work with a NOG. The KEGG ID of the gene encoding the protein is located to the right of each row, followed by the NOG with its functional category and function. Each column represents one of the 50 selected classes. The color inside the heatmap reflects the degree of distribution of a particular NOG in each class, with red representing complete distribution in all selected organisms of that class and light yellow representing absence in all selected organisms of that class. Source data are provided as a Source Data file.

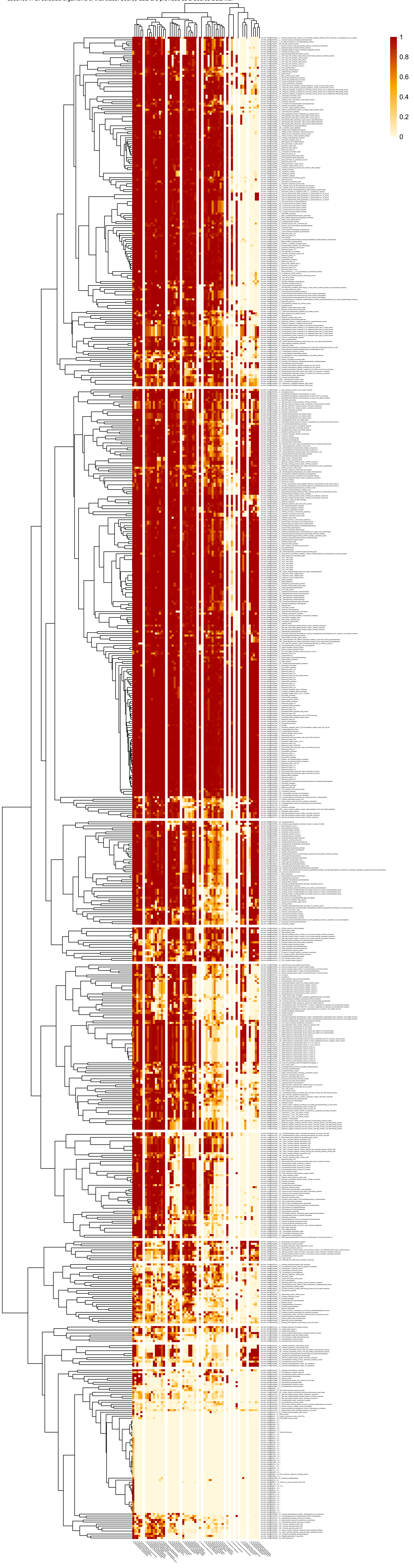

Figure S4. Complete phyletic distribution of *E. coli* essential genes. NOG heatmap according to the EggNOG database annotations for the *E. coli* essential gene set. Each row represents the essential genes reported by Goodall *et al.* with a NOG. The GenBank ID of the gene encoding the protein is located to the right of each row, followed by the NOG with its functional category and function. Each column represents one of the 50 selected classes. The color inside the heatmap reflects the degree of distribution of a particular NOG in each class, with red representing complete distribution in all selected organisms of that class and light yellow representing absence in all selected organisms of that class. Source data are provided as a Source Data file.

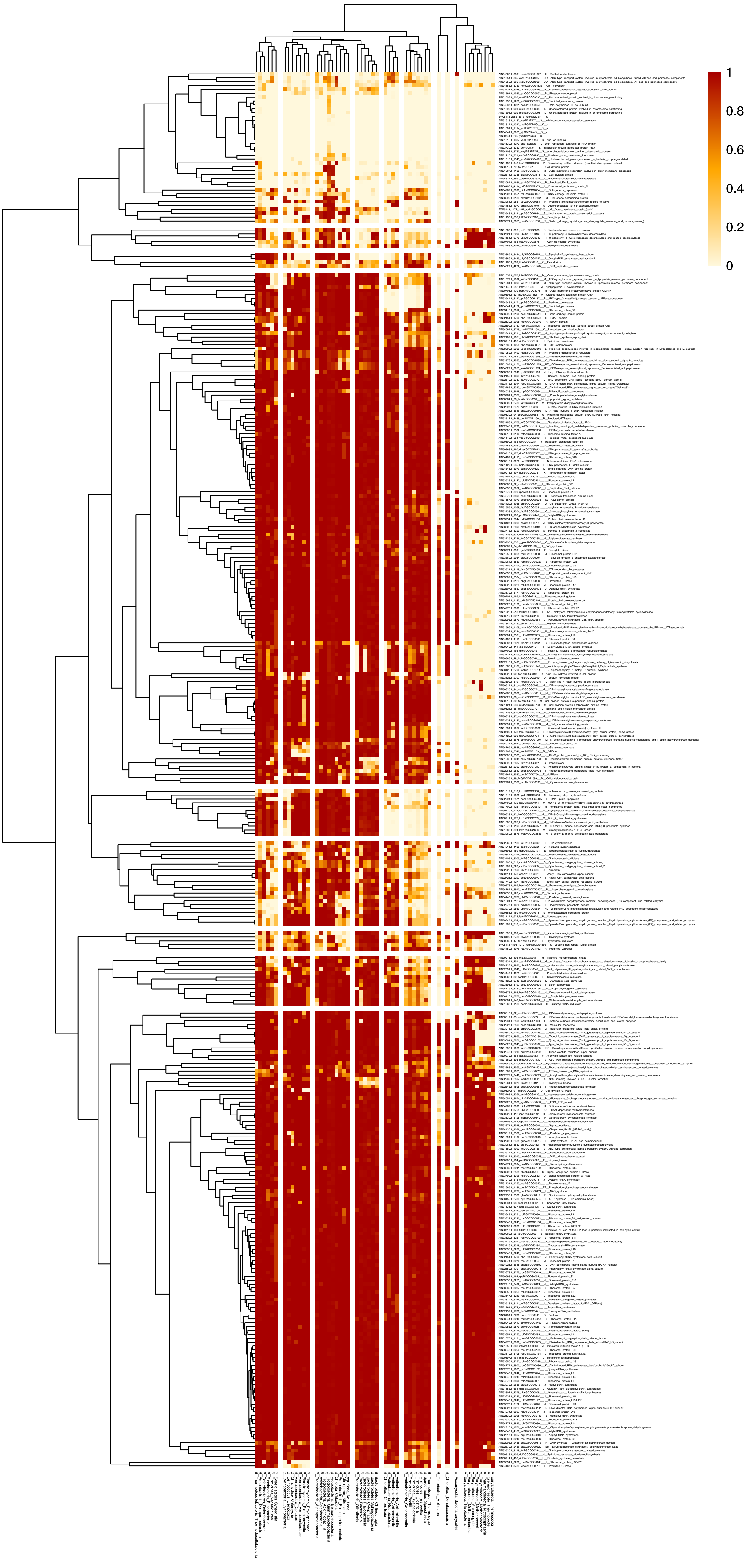

Figure S5. Deletion mutant checking PCR. a) PCR using primers annealing upstream and downstream to the flanking regions used as homologous sequences to obtain the deletion mutants. Expected sizes are indicated in bp in panel b. Primers used are shown in Sup. Table 4. L = DNA molecular weight ladder (Kbp) M = Mutant; Wt = Wild type; P = Plasmid; - = Negative control (H_2O). * Amplicon was sequenced to verify the PCR due to the similar size expected.

a

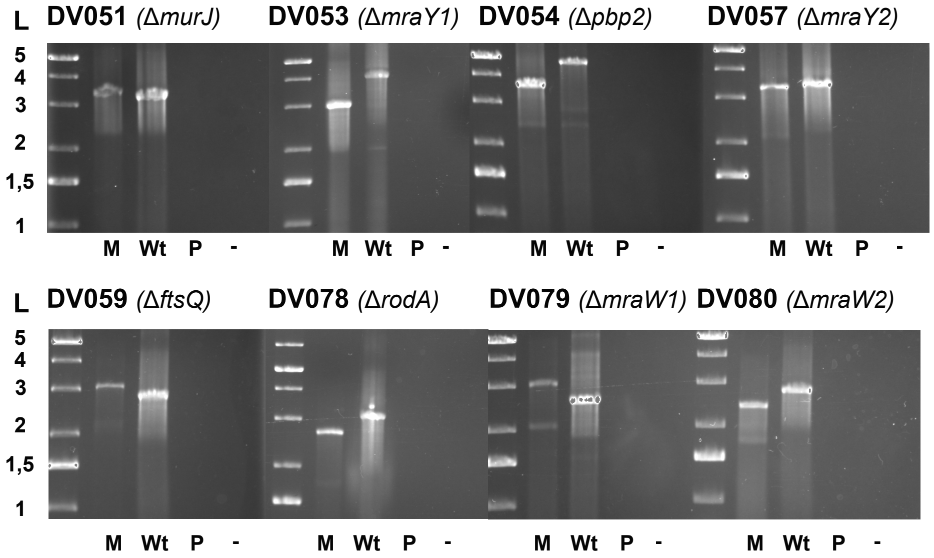

b

Strain	Mutant	Wild type	Plasmid
**DV051**	3310	3137*	na
**DV053**	3040	4052	na
**DV054**	3533	4553	na
**DV057**	3228	3265 *	na
**DV059**	2934	2656	na
**DV078**	2625	3073	na
**DV079**	2907	2539	na
**DV080**	2343	2728	na
