# Supplementary material for: Essential gene complement of Planctopirus limnophila from the bacterial phylum Planctomycetes
Source: Nat Commun. 2023 Nov 9;14:7224. doi: 10.1038/s41467-023-43096-3 (PMC10632474; doi:10.1038/s41467-023-43096-3)
Supplement: Supplementary file 3 — Description of Additional Supplementary Files [file 41467_2023_43096_MOESM3_ESM.pdf]

Description of Additional Supplementary Files:

Supplementary Data 1: Genome locations of unique transposon insertion events in .bed format.

Supplementary Data 2: List of genes classified as essential, not essential or unclear by TraDIS and sliding window.

Supplementary Data 3: Differences in essentiality with different cut-offs. Only the locus tag with at least one modification is being provided. The rest remains the same as in Dataset S2 despite the cut-off changes.

Supplementary Data 4: Genes with “domain” essential.

Supplementary Data 5: Orthologs with E. coli.

Supplementary Data 6: Conservation of P. limnophila proteins.

Supplementary Data 7: Paralogs in P. limnophila.

Supplementary Data 8: List of organisms used in the conservation analysis.

Supplementary Data 9: List of organisms used in the phyletic distribution.
